# Respect the synchrotron beam strength: how to model it, measure it and mitigate it for various scientific fields

**DOI:** 10.1107/S1600577521008328

**Published:** 2021-08-13

**Authors:** John R. Helliwell

**Affiliations:** a Science and Engineering Research Council (now Science and Technology Facilities Council), Daresbury Laboratory, Warrington WA4 4AD, United Kingdom

**Keywords:** extremely bright SR sources, materials science, macromolecular crystallography

## Abstract

Extremely bright synchrotron radiation sources give extremely strong intensities at the sample. Lawrence Bright *et al.* (2021) [*J. Synchrotron Rad.* (2021), **28**, 1377–1385] dive into the details for materials science. I offer a Commentary including a historical context.

It was 1983 when I was a beamline scientist at the UK’s SRS responsible for Protein Crystallography Stations 7.2 (bending magnet) and 9.6 (superconducting high field wiggler, 5 T) and I was on the European Synchrotron Radiation Project (ESRP) Working Group for Biology chaired by Dr Joan Bordas. We had just received the spectral emission specifications from the ESRP Project Leader Professor Bronislaw Buras based at CERN in Geneva. The spectral brightnesses and fluxes of the various ESRP sources, especially the undulators, showed colossal gains over what we had at the SRS. At SRS 9.6 I had already insisted to the engineers involved on having a straight-through beam option. The melting of lead by the white beam (Fig. 1 ▸) was a vivid effect to show us what we had at our disposal. It was one of the first ever synchrotron source wigglers. Duly concerned I measured the temperature rise in a sample of water in one of our standard protein crystal mounting glass capillaries, which (from memory) was about 1°C. The details of my test were very similar to those given in Fig. 11 of Cheng & Caffrey (1996 ▸) who used CHESS 13.6 keV X-rays of 2 × 10^10^ photons s^−1^ into a beam of size 0.3 mm and who reported a 0.16 K temperature rise. Therefore beam heating even on a second-generation source wiggler was not to be an anxiety for us as facility provider.

But, the beam strengths from the first ever third-generation source were far more (by up to ∼1000 more photons per second per mm^2^ into beam sizes of about 100 µm, instead of SRS 9.6 of ∼1 × 10^11^ 12 keV photons into a focus with our optics of ∼1 mm × 0.5 mm). Another major feature of the ESRP specifications was that this was a 5 GeV ring (ESRF was implemented at 6 GeV so that the undulator emission was better matched to the requested Mossbauer experiments). There were to be copious quantities of higher-energy photon fluxes to be available, *i.e.* we could consider quite easily working with 0.5 Å X-ray wavelength instead of typically 0.9 Å wavelength on SRS 9.6 and 1.488 Å on SRS 7.2 (both instruments were fully tunable for the anomalous dispersion-based studies).

Daresbury despatched me to work with Roger Fourme at the LURE synchrotron to consider these machine specifications in detail, namely their potential and yet also the possible problematic barriers to exploitation. I slept in the LURE users’ dormitory cabin which I had sole use of as it was a shutdown; I recall that eating in Orsay on a Sunday evening did not offer much by way of opportunity. Ultimately I presented our report (Helliwell & Fourme, 1983 ▸) to Professor Buras at CERN. We were actually very optimistic for macromolecular crystallography at the future ESRF. The calculations (Helliwell, 1984 ▸) showed that, in the worst case, the adiabatic model for the sample beam heating was serious but manageable. Specifically we proposed a new sample mounting to effect the best possible heat conduction away from the protein crystal. This comprised a crystal attached by grease to a copper stalk and surrounded by helium (Fig. 2 ▸). The grease would also protect the crystal from dehydration. Copper and helium were chosen for their high thermal conductivity. Later, at Daresbury and at other synchrotron sources we adopted the cryo-cooling methodology of Haas & Rossmann (1970 ▸), encouraged especially by Hakon Hope and Ada Yonath for realizing successful ribosome crystallography measurements (Hope, 1988 ▸). Room-temperature diffraction measurements fell out of fashion and our crystal sample mounting protocol dimmed from view.

New third-generation sources steadily appeared, as have a succession of lower emittance, higher spectral brightness, machine upgrades. After 17 years a new sample heating publication appeared (Kuzay *et al.*, 2001 ▸) and they even gave me a mention in their Contents’ page Synopsis, although not quite how I would appreciate it:

*‘Improved thermal models that include convection are developed which replace Helliwell’s adiabatic approximation. Temperature rises of 6 K are calculated for a cryocooled 100 micron thick crystal and of 18 K for a room-temperature air-cooled 1 mm thick crystal for an 8 keV 10^13^ photons s^−1^ mm^−2^ beam. The importance of internal heat conduction within the crystal is also carefully examined.’*

Apparently I was to be replaced rather than built upon.

I presented the different aspects of the Helliwell & Fourme (1983 ▸) ESRP internal report in the journals’ literature firstly in Helliwell (1984 ▸). The beam heating calculations had of course provided a basic ‘worst case’ adiabatic model. Kuzay *et al.* (2001 ▸) initiated the modelling of heat transfer from the sample, what I called an isothermal (*i.e.* steady state) model. We had of course immediately proposed an experimental solution, as mentioned above (Fig. 2 ▸). Further modelling studies, and measurements, followed [such as Snell *et al.* (2007 ▸)]. All these, including Kuzay *et al.* (2001 ▸), restricted themselves to modelling the case of a cryocooled protein crystal sample. An important exception was the extensive studies by Cherezov *et al.* (2002 ▸) who were constrained in their structural studies of lipid membranes and mesophases to room temperature.

Lawrence Bright *et al.* (2021) ▸ now lead the way for considering materials science and their samples’ beam heating. They provide as context for their initiative a full citing of the macromolecular crystallography beam heating papers, to my knowledge [except the Cherezov *et al.* (2002 ▸) studies] and a selection of the radiation damage papers. This latter is a much bigger literature thanks to the major efforts of the International Symposia on Radiation Damage in Macromolecular Crystallography [see, for example, Garman & Weik (2015 ▸)]. As part of the proposed ESRP and proposed New Rings at the Stanford Synchrotron Radiation Laboratory initiative led by Professors Keith Hodgson and Herman Winick, we also made evaluations of overall and specific (disulfide bond breaking) radiation damage (Hedman *et al.*, 1985 ▸; Helliwell, 1988 ▸).

The context for Lawrence Bright *et al.* (2021 ▸) is that the ESRF’s Extremely Bright Source (EBS) upgrade has been completed and even greater spectral source brightnesses duly realized at the turn of the year. They certainly respect their new ESRF EBS synchrotron beam strength. They offer their own ways of how to model it and measure it with different test samples relevant to materials science. They also offer ways to mitigate it. More than that, they suggest ways for experimenters to validate their measurements, learning from those experimental situations where experimenters should be concerned. As for the macromolecular crystallographers, the paper of Halle (2004 ▸) on cryo structural artefacts is encouraging us to more firmly establish when those artefacts might be serious. Our proposed sample mount (Fig. 2 ▸) may yet come into fashion. Also the helium gas would be in an enclosure allowing control of the crystal’s temperature to be at 37°C, *i.e.* physiological for mammals. That temperature would not allow much headroom as the protein melting temperature for mammalian proteins is around 60°C. The alternative solution to Fig. 2 ▸, imported from the XFEL facilities, of serial crystallography of course implicitly assumes that every sample is identical. In a recent study I was thwarted in my detailed analyses because I merged the cryodiffraction data of two apparently identical crystals; I only made headway when I analysed them separately (Govada *et al.*, 2021 ▸). Meanwhile, cryocrystallography continues to prove very effective in synchrotron facility measurement pipelines and with more and more remote working, as well as mitigating beam heating. These cryo crystal structures, the vast majority in the Protein Data Bank, have also dominated the training set though for prediction of 3D structures using deep learning from amino acid sequences (Helliwell, 2020 ▸).

Finally, I note that in Lawrence Bright *et al.* (2021 ▸) the adiabatic approximation has enjoyed a revival with six detailed mentions, and with much more besides. Suffice to say, Lawrence Bright *et al.* (2021 ▸) is I think an important paper.

## Figures and Tables

**Figure 1:**
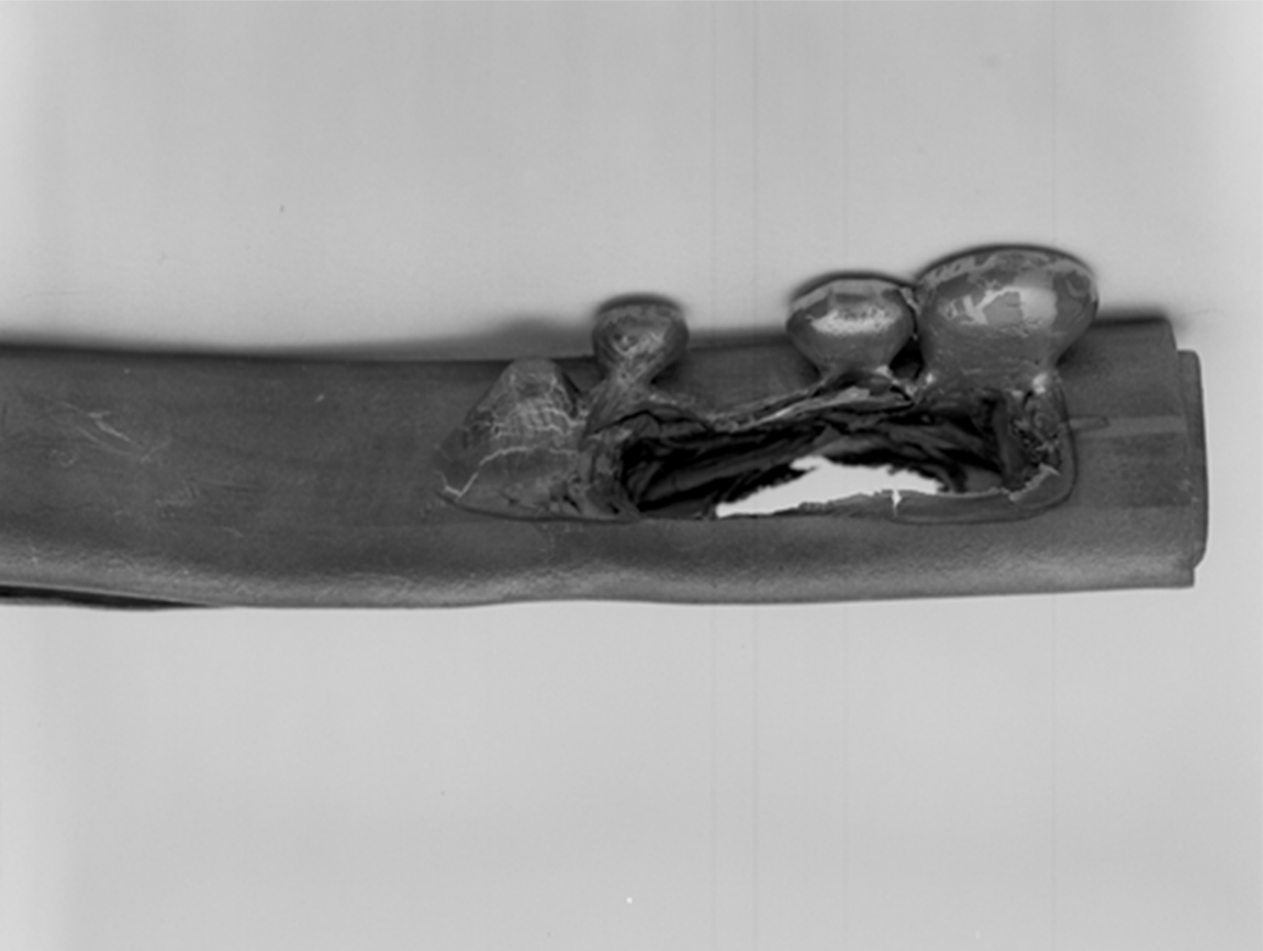
A piece of lead, aligned horizontally, at the SRS 9.6 melts due to the white beam.

**Figure 2:**
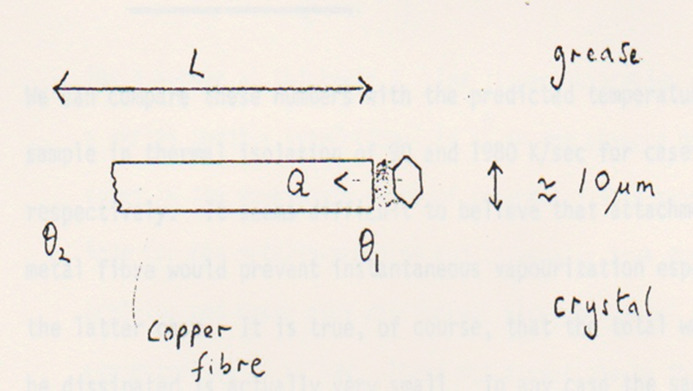
A strategy to control crystal sample heating was offered for room-temperature X-ray diffraction measurements at the exceptionally large ESRF X-ray intensities that were planned (Helliwell & Fourme, 1983 ▸).
